# Synchronous supratentorial and infratentorial oligodendrogliomas with incongruous *IDH1* mutations, a case report

**DOI:** 10.1186/s40478-021-01265-9

**Published:** 2021-09-29

**Authors:** Alexander H. C. W. Agopyan-Miu, Matei A. Banu, Michael L. Miller, Christopher Troy, Gunnar Hargus, Peter Canoll, Tony J. C. Wang, Neil Feldstein, Aya Haggiagi, Guy M. McKhann

**Affiliations:** 1 Columbia University Vagelos College of Physicians and Surgeons, New York, USA; 2 Department of Neurosurgery, Columbia University Irving Medical Center, New York, USA; 3 Department of Pathology and Cell Biology, Columbia University Irving Medical Center, New York, USA; 4 Department of Radiation Oncology, Columbia University Irving Medical Center, New York, USA; 5 Department of Neuro-Oncology, Columbia University Irving Medical Center, New York, USA

**Keywords:** Multifocal, Low-Grade Glioma, IDH mutant, Supratentorial, Infratentorial

## Abstract

Infratentorial oligodendrogliomas, a rare pathological entity, are generally considered metastatic lesions from supratentorial primary tumors. Here, we report the case of a 23-year-old man presenting with a histopathologically confirmed right precentral gyrus grade 2 oligodendroglioma and a concurrent pontine grade 3 oligodendroglioma. The pontine lesion was biopsied approximately a year after the biopsy of the precentral lesion due to disease progression despite 4 cycles of procarbazine-CCNU-vincristine (PCV) chemotherapy and stable supratentorial disease. Histology and genetic analysis of the pontine biopsy were consistent with grade 3 oligodendroglioma, and comparison of the two lesions demonstrated common 1p/19q co-deletions and *TERT* promoter mutations but distinct *IDH1* mutations, with a non-canonical *IDH1* R132G mutation identified in the infratentorial lesion and a R132H mutation identified in the cortical lesion. Initiation of Temozolomide led to complete response of the supratentorial lesion and durable disease control, while Temozolomide with subsequent radiation therapy of 54 Gy in 30 fractions resulted in partial response of the pontine lesion. This case report supports possible distinct molecular pathogenesis in supratentorial and infratentorial oligodendrogliomas and raises questions about the role of different *IDH1* mutant isoforms in explaining treatment resistance to different chemotherapy regimens. Importantly, this case suggests that biopsies of all radiographic lesions, when feasible and safe, should be considered in order to adequately guide management in multicentric oligodendrogliomas.

## Introduction

Oligodendrogliomas represent approximately 5% of all glial tumors in the US adult population [13]. Those arising in the infratentorial compartment are rare as most arise in the cerebrum. Thus far, only a few isolated case studies have reported genetically confirmed, IDH mutant and 1p/19q-codeleted, infratentorial oligodendrogliomas [3, 4, 6, 10, 13]. High-grade gliomas in adolescents and young adults (AYA, 15–25 years old) have been recently shown to harbor distinct molecular features compared to both the pediatric and adult population. In this particular age group, WHO grade may have limited utility whereas molecular subtypes have a major prognostic impact [19]. Interestingly, noncanonical *IDH1* mutations have been identified with a higher frequency in AYA gliomas compared to the adult patient population [19]. The role of distinct molecular features in selecting management strategies, however, remains unclear.

The simultaneous presence of multiple lesions, especially lesions with supratentorial and infratentorial components, is a rare occurrence in gliomas of all type [20]. Concurrent lesions are classified as either multifocal or multicentric based on radiographic and/or pathologic features. Multifocal lesions are those that either demonstrate a path of contiguous hyperintensity on T2 MR images, or if the pattern of dissemination can be explained by spread along white matter tracts, CSF, or local spread from satellite lesions [14]. Multicentric lesions, on the other hand, cannot be described by the previous features and includes those lesions separated by time, termed metachronous lesions [14]. Given the rarity of synchronous lesions, little is known about their underlying molecular/genetic relationships. Some studies proposed independent gliomagenesis events. For example, Lee et al. [12] suggest that multifocal/multicentric tumors are seeded from geographically segregated, distinct tumor clones given the few shared genetic alterations they observed between multifocal/multicentric tumors. On the other hand, multicentric or multifocal lesions might represent intracranial metastasis of a primary focus, with a common clonal precursor [2, 15]. Several recent studies have suggested potential mechanisms through early divergence and parallel evolution eventually leading to genetically distinct lesions [1, 9]. Subclonal drivers with late occurrence rather than founder events were believed to drive the evolution of such lesions [1]. In this regard, the role of early events during gliomagenesis, such as *IDH1* mutations, in driving multicentric gliomas remains elusive. The clinical utility of classifying multiple concurrent gliomas as either multifocal or multicentric remains unclear. In the absence of specific guidelines, management largely resembles that of solitary lesions despite the worse prognosis associated with multifocal/multicentric gliomas [8, 14].

Here, we present a rare case of a 23-year-old man with synchronous, genetically confirmed right precentral grade 2 and pontine grade 3 oligodendroglioma. The two lesions harbored similar 1p/19q-codeletions and *TERT* mutations but have different *IDH1* mutations: a canonical *IDH1* R132H was identified in the precentral lesion while a noncanonical *IDH1* R132G mutation was identified in the pontine lesion. Importantly, the two lesions differed in their response to four cycles of procarbazine-CCNU-vincristine (PCV) chemotherapy. The case illustrates one possible pathogenesis of multifocal/multicentric oligodendrogliomas, via early subclonal divergence and parallel evolution, and suggests the possible importance of performing biopsies of all radiographically visible lesions, when safe and technically feasible, as it may impact subsequent treatment decisions.

## Case presentation

**Fig. 1:**
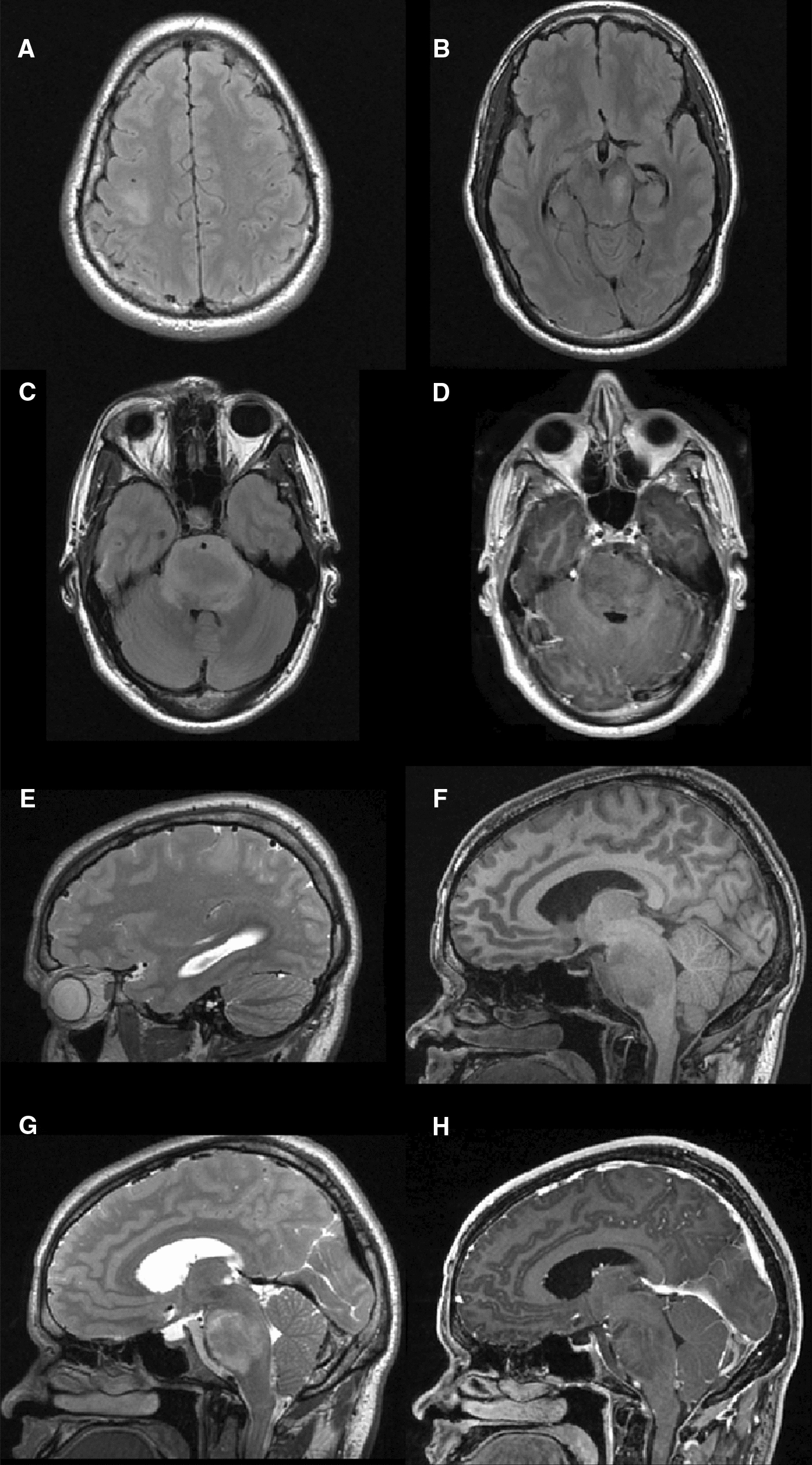
MRI of multifocal lesions on presentation. **A** Axial T2-FLAIR demonstrating a hyperintense round lesion, measuring 2.7 × 2.2 × 1.5 cm (CC × AP × transverse), in the precentral gyrus. Initial measurements prior to presentation at our institution: 2.2 × 1.7 × 1.5 cm. **B** Left greater than right hyperintensity in the cerebral peduncles on axial T2-FLAIR. **C** Axial T2-FLAIR demonstrates a heterogenous, hyperintense lesion in the pons, measuring 3.9 × 3.1 × 4.4 cm, with extension into bilateral cerebellopontine cisterns and middle cerebellar peduncles. Encasement of the basilar artery is also present with no obstruction of flow. Initial measurements prior to presentation at our institution: 3.5 × 3.1 × 4.3 cm. **D** Axial T1 post-contrast image of the pontine lesion demonstrating subtle patchy contrast enhancement. **E** Sagittal T2 image of the precentral lesion with compression of the central sulcus. **F** Large heterogenous, T1 hypointense pontine lesion extending from the floor the fourth ventricle to the prepontine cistern ventrally. **G** Pontine lesion as described with heterogenous hyperintensity on this sagittal T2 slice. **H** Subtle patchy enhancement is again appreciated within the pontine lesion on this T1 post-contrast sagittal slice

The patient, a previously healthy 23-year-old man, first presented to an outside hospital with complaints of intermittent diplopia, tinnitus and occipital pain. Due to persistent headache, he underwent an MRI scan which revealed synchronous, non-enhancing, T2/FLAIR hyperintense lesions in the right precentral gyrus and pons as well as increased signal along the right 8th cranial nerve. Initial serial scans were stable and the patient remained in stable condition during that time but eventually presented to our institution 8 months later for a second opinion. By this time, he developed headaches, mild photophobia, left-sided weakness, and left facial numbness. Initial in-house imaging at the time demonstrated a 3.9 × 3.1 × 4.4 cm heterogenous, but primarily T1 hypointense, T2 hyperintense, infiltrating, and mildly expansile pontine lesion with minimal patchy contrast enhancement and diffusion restriction that was larger in size compared to imaging obtained from the outside hospital. Extension into the floor of the fourth ventricle, compression of the prepontine cistern, and obstructive hydrocephalus was noted. Extension into the middle cerebellar peduncles and encasement of the basilar artery was also noted. MR spectroscopy revealed elevated choline peak and reversal of the choline/NAA ratio, and was overall consistent with a glial tumor. A second infiltrating and expansile, non-enhancing lesion, measuring 2.7 × 2.2 × 1.5 cm, in the right precentral gyrus with similar imaging characteristics to the pontine lesion was also seen and noted to have increased in size compared to imaging studies obtained from the outside hospital (Fig. 1). MR perfusion demonstrated low relative cerebral blood volume and flow values, suggestive of a low-grade glioma.

A ventriculoperitoneal shunt was placed to treat the patient’s hydrocephalus. He subsequently underwent an awake craniotomy with sensorimotor mapping for an excisional biopsy of the right peri-rolandic lesion. A subtotal resection was carried out, to avoid inducing a left-hand sensory deficit.

**Fig. 2:**
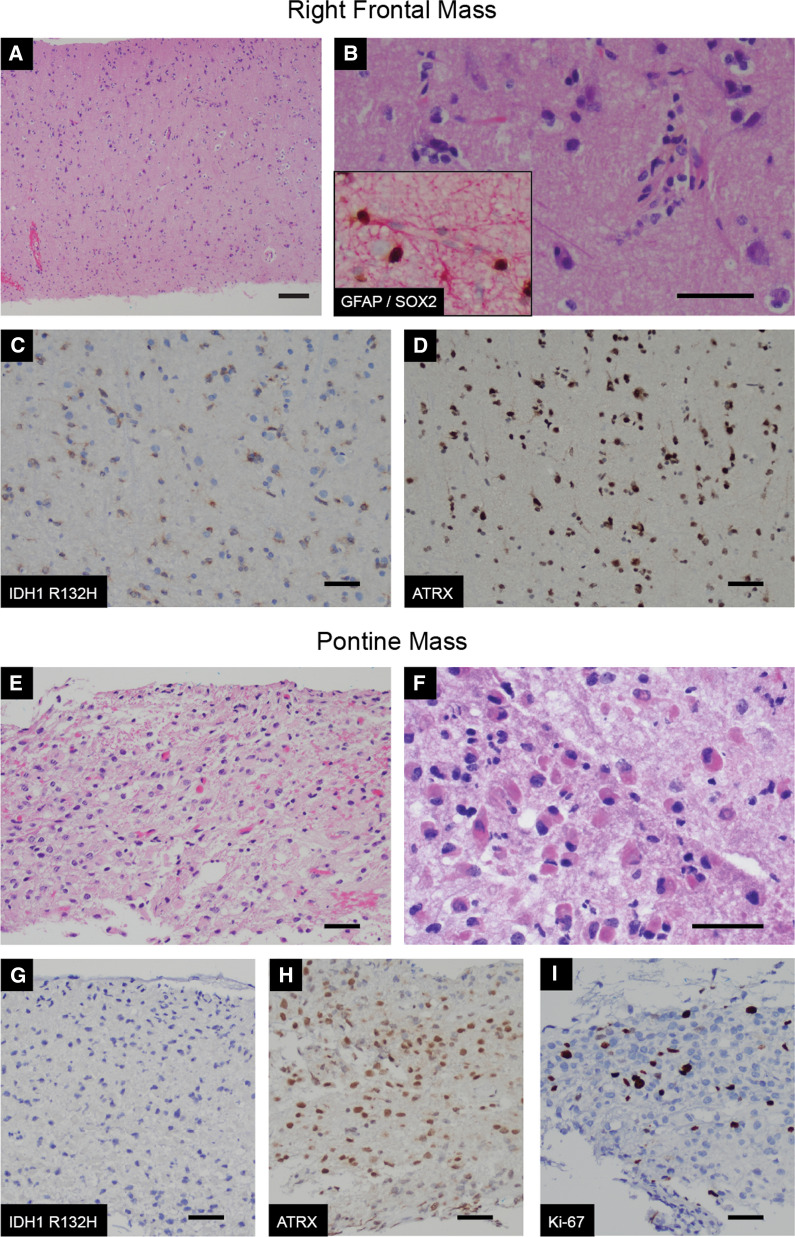
Genetically divergent multifocal glioma involving cerebrum and brainstem. **A**–**D** Biopsies of the cortical mass revealed a diffusely infiltrating glial neoplasm with perineuronal satelitosis and perinuclear clearing (**A**, 4×; **B**, 400×). The neoplastic cells expressed SOX2 (brown chromogen) and GFAP (red chromogen) (**B** **inset**, 200×), and harbored the IDH1 R132H onco-protein (**C**, 200×) with retained nuclear expression of ATRX (**D**, 200×). **E**–**I** Biopsies of the pontine mass revealed a diffusely infiltrating glial neoplasm with increased cellularity and prominent mini-gemistocytic cytomorphology (**E**, 200×; **F**, 400×). As opposed to the cortical mass, the pontine mass lacked the IDH1 R132H onco-protein (**G**, 200×) however nuclear expression of ATRX was similarly retained (**H**, 200×). Ki-67 index was increased in the pontine biopsy – a representative image is provided (**I**, 200×). (Scale bar = 50 um.)

Final pathological analysis of the biopsied right peri-rolandic lesion demonstrated a low grade diffusely infiltrating glial neoplasm with no microvascular proliferation, necrosis, or mitotic activity. The Ki-67 proliferation index was modestly increased, labeling up to approximately 1.5% of tumor cells. The cells were positive for GFAP, SOX2, IDH1 R132H, and PDGFR-A by immunohistochemistry (Fig. 2), and ATRX staining was preserved. Rare cells showed weak p53 staining, and immunostains for H3 K27M and EGFR were negative. Targeted next generation sequencing panel (FoundationOne CDx, Foundation Medicine, Boston, MA) revealed the presence of a *TERT* promoter mutation (variant allele fraction, VAF, of 23%) and confirmed the *IDH1* R132H mutation (VAF of 19%), while consistent with immunostain result, no mutation in *H3F3A* was seen (Table 1) Further studies confirmed the presence of 1p/19q-codeletion via fluorescence in situ hybridization (FISH) and an unmethylated *MGMT* promoter by bisulfite-treatment PCR and melting curve analysis. Based on these findings, the final diagnosis was oligodendroglioma, IDH-mutant and 1p/19q-codeleted, WHO Grade 2.

**Table 1:** Comparison of genetic findings between the precentral and pontine lesion

Precentral	Pontine
IDH1 R132H by IHC and next gen sequencing	IDH1 R132G by next gen sequencing
1p/19q-codeleted by FISH	1p/19q-codeleted by FISH
TERT promoter mutation 146 C > T by next gen sequencing	TERT promoter mutation 146 C > T by next gen sequencing
ATRX preserved by IHC	ATRX preserved by IHC
Unmethylated MGMT by MGMT methylation promoter assay	Partial MGMT methylation by a MGMT methylation promoter assay
PDGFR-A positive	H3 K27M negative by IHC and next gen sequencing
H3 K27M negative by IHC and next gen sequencing	

Imaging a month later demonstrated a decrease in size of the ventricular system, a stable peri-rolandic lesion, and stable faint patchy enhancement within the pontine lesion that again demonstrated an interval increase in size compared to the initial scan obtained at the outside hospital. Despite these changes the pontine lesion was considered to likely be an oligodendroglioma given the pathology of the peri-rolandic lesion, albeit with features concerning for a grade 3 lesion on imaging. After a comprehensive multidisciplinary tumor board discussion considering the risks associated with biopsy of the brainstem lesion and the patient’s treatment preference, the decision was made to proceed with a standard PCV chemotherapy regimen alone. During chemotherapy, the patient’s course was complicated by mild peripheral neuropathy and asymptomatic thrombocytopenia necessitating a 25% dose reduction for the third cycle, and a further 25% dose reduction at the start of the fourth cycle in light of recurring thrombocytopenia. Overall, his clinical picture improved, with resolution of headaches, improvement in diplopia, and interval MRI scans up to 8 months out showed stable disease.

**Fig. 3:**
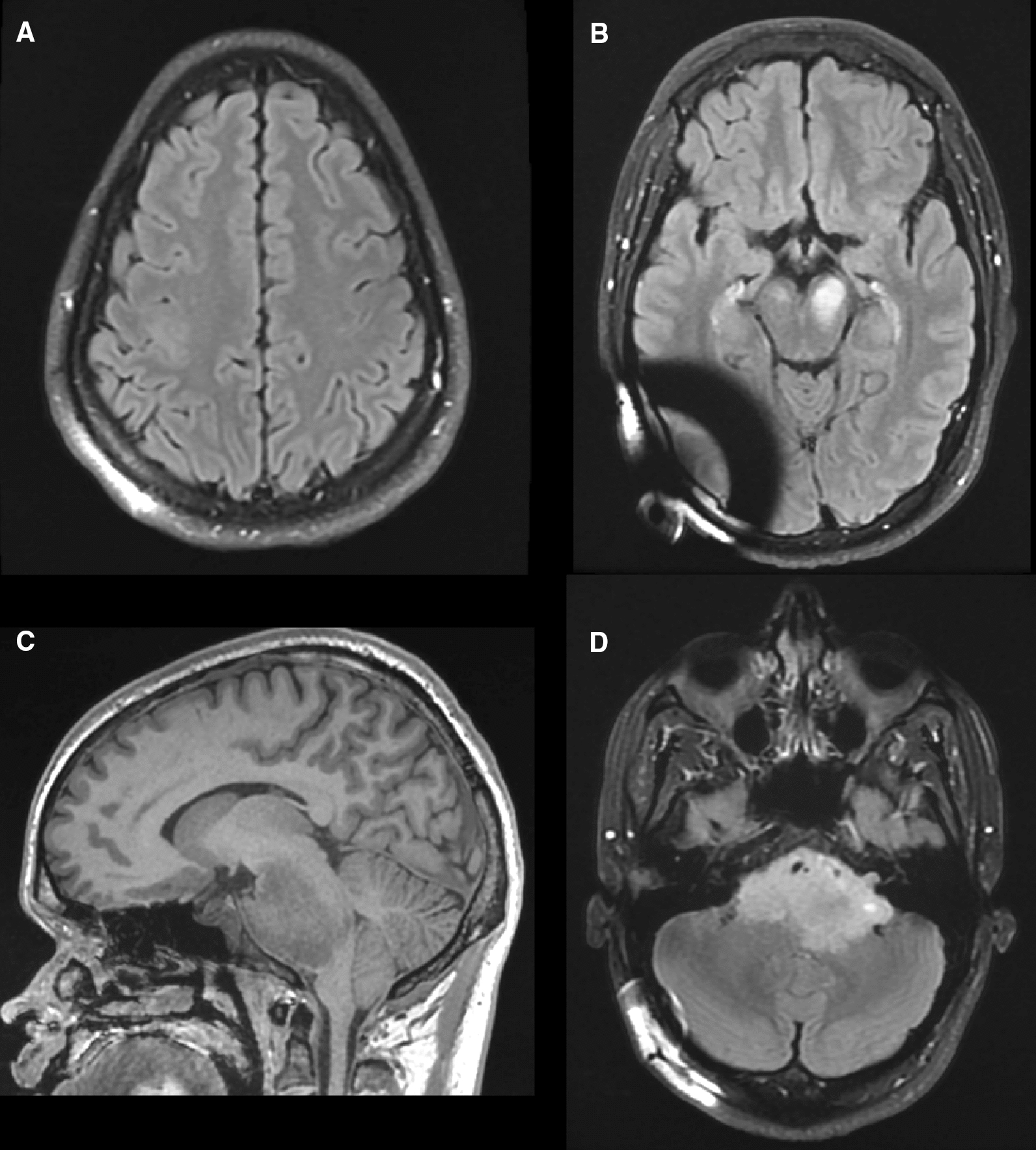
Follow up MRI following four cycles of PCV. **A** Hyperintense lesion of similar size and appearance in the precentral gyrus on axial T2-FLAIR. **B** Homogenous hyperintense lesions in the left and right cerebral peduncles on axial T2-FLAIR. **C** Large, primarily hypointense pontine lesion on sagittal T1 measuring 6.1 × 4.8 × 3.9 cm. Note herniation of the cerebellar tonsil. **D** Axial T2-FLAIR demonstrating new hyperintense foci along the left anterior base of the lesion that extends into the cerebellopontine cistern and internal auditory canal. Again, encasement of the basilar artery is noted but flow is patent

Imaging at 9 months after initiation of chemotherapy demonstrated new small foci of enhancement in the brainstem lesion, herniation of the cerebellar tonsils, and a stable cortical lesion (Fig. 3). The patient had now developed progressive suboccipital headaches. The decision was made to proceed with surgery for suboccipital decompression of the acquired Chiari malformation together with biopsy of the pontine lesion to guide further management.

Three small cores of tissue were obtained from the enhancing portion of the pontine lesion. Final pathological analysis of the biopsied brainstem lesion revealed a glial neoplasm of increased cellularity, nuclear pleomorphism, and proliferative activity with up to 3 mitotic figures in this small biopsy and a Ki-67 labeling index of nearly 10%. No tumor necrosis or microvascular proliferation was appreciated. Similar to the supratentorial tumor cells, these tumor cells were positive for GFAP, SOX2, and ATRX. However, IDH1 R132H was unexpectedly negative by immunohistochemistry. FISH confirmed the presence of 1p/19q-codeletion. Targeted next generation sequencing panel revealed a *TERT* promoter mutation (VAF 55%) identical to the peri-rolandic lesion and an *IDH1* R132G mutation (VAF 46%) not seen in the patient’s supratentorial tumor. No other mutations were identified on this laboratory-developed platform which includes assessment of *IDH1*, *IDH2*, *H3F3A* and *HIST1H3B* (Columbia Solid Tumor Panel, Personalized Genomic Medicine at Columbia University, New York, NY). The *MGMT* promoter was partially methylated (Table 1). Based on the overall findings, the final diagnosis was anaplastic oligodendroglioma, IDH-mutant and 1p/19q-codeleted, WHO Grade 3.

The patient tolerated the procedure well and experienced complete resolution of his headaches. Post-operatively he underwent radiation therapy to the brainstem lesion, 54 Gy in 30 fractions, concurrently with systemic Temozolomide chemotherapy for 6 weeks followed by adjuvant Temozolomide, which he continues to this date. He continues to improve clinically, with a KPS of 80 and is able to ambulate independently at the time of this report. He initially developed diplopia which has remained stable since initiation of chemoradiotherapy. He has been off steroids. The most recent MRI at the time of this report, at 27 months post diagnosis, revealed decrease in size of the pontine lesion with near complete resolution of the peri-rolandic lesion and no new supratentorial lesions. Notably, the peri-rolandic lesion showed radiographic response prior to initiation of Temozolomide suggesting PCV chemotherapy benefit.

## Discussion

Oligodendrogliomas are relatively rare, well-differentiated neuroepithelial tumors that more commonly occur in the cerebral hemispheres compared to the infratentorial space [10]. We present here a unique case of synchronous supratentorial and infratentorial oligodendrogliomas in a young adult patient with no known predisposing germline mutations. The stark difference in response to four cycles of PCV between the cortical and pontine lesions necessitated the biopsy of the latter and provided the opportunity to compare genetic and histopathologic characteristics. Such studies can provide insight into the origin of distinct tumors and the evolutionary trajectories of IDH-mutant oligodendrogliomas. The most striking finding from the pontine biopsy was the presence of a non-canonical *IDH1* R132G mutation that differed from the canonical *IDH1* R132H mutation encountered in the supratentorial lesion. This finding suggests two possible avenues of pathogenesis.

One hypothesis is the presence of two distinct, multicentric tumors that evolved synchronously and independently of each other. Greater rates of non-canonical *IDH1* mutations have been reported in infratentorial gliomas compared to supratentorial gliomas, and in lower grade gliomas overall [5, 16, 18, 23]. Given that both canonical and non-canonical *IDH1* mutations likely represent early clonal mutations that may even precede 1p/19q-codeletions [11, 23–25], the divergent *IDH1* mutations may suggest the synchronous development of two independent low grade gliomas whose distribution is consistent with the reported anatomic distribution and prevalence of mutant *IDH1* isoforms thus far.

An alternative hypothesis is that the infratentorial lesion is in fact a metastatic lesion originating from the supratentorial tumor. In this case, the distinct *IDH1* mutations may indicate an evolutionary trajectory that led to a more aggressive subclone, capable of metastatic spread. Therefore, the *IDH1* R132G mutation may mark a unique subclone with increased ability to metastasize via corticospinal tract fibers or CSF. Furthermore, it is important to note that the biopsies were separated in time and therefore may in fact capture the temporal evolution of different subclones. The *IDH1* R132G mutant cells may have had increased chemoresistance at baseline and may have become the predominant population after PCV chemotherapy secondary to a subclonal selection process.

In future studies of multicentric gliomas, synchronous longitudinal biopsies coupled with in-depth genetic sequencing could help us infer the timing of clonal separation between the R132G and R132H populations during tumorigenesis. In this case, we are unable to definitively draw conclusions about the pathogenesis of these spatially distributed oligodendrogliomas but provide a roadmap for future studies. Furthermore, we provide evidence that distinct lesions with distinct mutations may have different sensitivities to various chemotherapy and radiation regimens, therefore having important clinical implications.

The role of *IDH1* mutations in predicting response to chemotherapy for oligodendrogliomas has been difficult to establish, in contrast to the well-established predictive value of 1p/19q-codeletions in predicting sensitivity to PCV. The presence of IDH1 mutations in conjunction with 1p/19q-codeletions may indicate a positive prognosis in oligodendroglioma patients treated with adjuvant PCV following radiation therapy [7, 21, 22, 26]. However, it is unclear how many patients in these studies had infratentorial lesions or non-canonical *IDH1* mutations. Furthermore, there are few studies comparing the predictive and prognostic value of non-canonical *IDH1* mutations to the canonical *IDH1* R132H mutation. Enzyme kinetics have been shown to differ between mutant isoforms, and isoforms that produce greater amounts of D-2-hydroxyglutarate, like the R132G isoform, are disadvantaged by the toxicity associated with the metabolite’s buildup [17]. This mechanism may underlie the proposed favorable prognostic associations with non-canonical mutations in oligodendrogliomas [23]. Thus, determining whether the divergent *IDH1* mutations played a role in the differential treatment response is difficult. It is also important to note that, after discussion of available treatment options, a chemotherapy only as opposed to radiotherapy plus adjuvant chemotherapy treatment plan was chosen to respect patient preferences. The addition of radiation later in the course with subsequent improved control may indicate the need for more aggressive multimodality approaches in such lesions.

With respect to overall management, we believe that this case highlights the importance of (1) performing biopsies on all radiographically visible lesions if safe to do so, (2) performing biopsies of treatment resistant lesions, even later in the treatment course and (3) sequencing the *IDH1* exon if initial immunohistochemical staining is negative. Even if one subscribes to a multicentric or multifocal theory of pathogenesis, this case shows that there can be notable heterogeneity between foci, and this heterogeneity may impact treatment response [12]. Further, Visani et al. [23] note that a significant proportion of low-grade gliomas in their case series would have been misclassified by WHO 2016 criteria if an investigation only for the canonical R132H mutation was performed. While there is little evidence to suggest a difference in prognostic or predictive value between canonical and non-canonical *IDH1* mutations at present, knowing the precise genetic makeup of multicentric or multifocal lesions can guide treatment decisions as more data is uncovered.

## Conclusion

We present here a rare case of multifocal high grade oligodendroglioma with supratentorial and infratentorial lesions, distinct *IDH1* mutations, and differential response to PCV chemotherapy. This case illustrates one potential evolutionary trajectory of multicentric oligodendrogliomas and provides insight into best management practices for these rare tumors. We speculate that the infratentorial lesion may be a metastatic, higher grade lesion arising from the supratentorial mass, and that the non-canonical *IDH1* mutation marked sub-clones with increased tumor invasiveness and/or chemoresistance which diverged early on in tumorigenesis. Alternatively, the supratentorial and infratentorial oligodendrogliomas may represent completely separate, synchronous lesions with distinct genetic alterations. Irrespective of time course in gliomagenesis, this case report highlights the importance of performing individual biopsies of all radiographic lesions, both for therapeutic decisions and for prognostication. Overall, infratentorial low-grade oligodendrogliomas remain a rare and poorly understood pathological entity. Further studies are needed to gain more insight into gliomagenesis and best management practices.

## Data Availability

Data sharing is not applicable to this article as no datasets were generated or analyzed during the current study.

## Abbreviations

PCV: Procarbazine-CCNU-Vincristine
AYA: Adolescent and Young Adult
IDH: Isocitrate Dehydrogenase
